# Cultural competence and curricular design: learning the hard way

**DOI:** 10.1007/s40037-018-0428-7

**Published:** 2018-05-07

**Authors:** Mary Kate Worden, Nassima Ait-Daoud Tiouririne

**Affiliations:** 1 Department of Medical Education, University of Virginia School of Medicine, Charlottesville, VA USA; 2 Department of Psychiatry and Neurobehavioral Sciences, University of Virginia School of Medicine, Charlottesville, VA USA

**Keywords:** Cultural humility, Cultural competency, Active learning

## The story

Since the Liaison Committee on Medical Education formalized a standard for cultural competence in 2000 [1], medical schools have been challenged with how to effectively teach and assess this topic in the undergraduate medical curriculum. In the University of Virginia School of Medicine UME pre-clerkship curriculum, an instructor lectured annually on cultural competence; however, many students considered the topic ‘soft’ by comparison to anatomy, physiology and other biosciences and few attended. The educational effectiveness of the lectures was difficult to determine, given the inadequacy of the multiple choice question format for assessing students on cultural competence with pre-clerkship exams, and the absence of any other form of meaningful assessment.

When the lecturer retired, the course faculty considered how best to continue teaching this topic, given the importance of cultural competence/cultural humility for eliminating healthcare disparities and providing high-quality patient care [2]. While we recognized that the third year clerkship students who were caring for patients might be more likely to value formal curricular attention to cultural topics, we decided that eliminating the topic from the pre-clerkship curriculum might send an unintended ‘hidden curriculum’ message that the biosciences are of overwhelming importance in preparing for a career in medicine. In addition, we were hopeful that our pre-clerkship OSCEs might ultimately prove to be useful tools for assessing the impact of our educational efforts around the cultural competency/cultural humility theme.

As our pre-clerkship curriculum emphasizes active learning [3], our instructors have become experienced in writing Team Based Learning™ [4] exercises on a wide variety of bioscience and ethical topics. Several members of the course faculty collaborated to design a TBL exercise on the topic of culture. The pre-class assignment for this exercise included two readings. One described two different approaches for communication in the practice of culturally competent medicine: the LEARN model of Berlin and Fowkes [5] and the exploratory model developed by Kleinman and colleagues [6]. The other was a board-review style summary of cultural characteristics of four ethnic minority subcultures that students were likely to encounter in the US healthcare system. The in-class exercises focused on several patient vignettes in which cultural difference posed a barrier to medical diagnosis or treatment. Each of the patient vignettes was drawn from the professional and personal experiences of the instructor, an immigrant to the US who was born and raised in Africa, practises medicine with a multicultural patient population, and speaks several languages. For each vignette, students were asked what the healthcare provider should do or say.

As TBL exercises require teamwork, we required attendance of all 156 first year medical students at this session, of whom >20% self-report as racial and ethnic populations that are underrepresented in medicine. We anticipated that converting the lectures to TBL format would serve our twin curricular goals of stimulating active learning in the classroom through discussion and raising student awareness about how best to navigate cultural barriers that might compromise high quality patient care.

## Surprising outcomes

The first year we ran this TBL exercise we were surprised that student reaction was vehemently negative; the class discussion veered off the topic of the vignettes and into emotionally charged territory that empowered some students to dominate the discussion and intimidated others. Some students felt insulted by the fact that we required their attendance to a session focused on cultural topics; they felt their own prior life experiences in a ‘post-Obama’ age made them sufficiently culturally competent to be able to provide high-quality patient care. (This criticism had not been made in prior years by the minority of the students who ‘opted-into’ attending the lectures). Some thought the instructor had committed an act of bias in assigning the readings, which they felt listed negative stereotypes related to culture and race. Many felt that treating all patients ‘equally’ and similarly regardless of culture would be the best course of action in all cases, and suggested it would be wrong to assume *a priori* that culture plays a role in a patient’s experience with health, illness, and the healthcare system, as our TBL vignettes implied.

To our astonishment, some argued the vignettes seemed inauthentic, because instances in which healthcare hinged on a cultural issue must be rare. Sadly, others felt that as ethnic or racial minorities themselves, their thoughts and experiences were not being heard in the conversation and emailed the course director to say that they were afraid to speak up in opposition to their classmates.

After discussing the student concerns, we modified the TBL exercise in several ways over subsequent years. First, we introduced the session with a brief talk by the Associate Dean for Diversity to explain that all healthcare providers can benefit from learning more about the cultural barriers to patient care and practising their approach to them, no matter how extensive their prior life experiences with individuals from different races and cultures. Second, we changed the learning goal from ‘cultural competence’ to ‘cultural awareness’ to highlight that *no one* is ever completely competent in a continuously changing landscape of cultures and human interactions. Third, we kept the summaries of the LEARN and Kleinman models but replaced the other pre-class reading with a white paper in which members of four national ethnic minority psychological associations describe their own cultures in their own words and highlight culture specific views of health and healing [7]. Fourth, we invited other instructors with different cultural backgrounds to help moderate the discussion and emphasize that cultural barriers to healthcare are neither rare nor best ignored in service of ‘treating everyone equally’. Finally, we redoubled our efforts to ensure all students could voice their thoughts in class by encouraging and involving as many as possible in the large group discussion.

While student reaction to the Culture TBL was more subdued in subsequent years, it remained strongly negative. Students continued to question the importance of culture in the clinical encounter, challenged the validity and content of the white paper that replaced the second reading (although it had been authored by members of minority groups) and seemed doubtful about the cultural knowledge and experience of the instructors, although the instructors were all experienced physicians and members of minority groups themselves.

## Lessons learned

The biggest lesson we learned from this experience was that the topic of culture stimulates a variety of strong emotional triggers for students. We had assumed that our faculty’s considerable success in converting bioscience and ethics lectures into TBL exercises would transfer to the topic of race and culture, and help make this a meaningful and practical component of the curriculum. In general, our students appreciate that TBLs challenge them to reach consensus on difficult topics and they value the peer-to-peer teaching moments that TBLs foster. Our students also recognize the educational benefits of struggling with questions that do not lend themselves to simple answers. However, our course faculty learned the hard way that the topic of culture can derail discussions, sow divisions between classmates and ultimately hinder student learning.

Upon reflection, we recognize that the topic of cultural competence likely has the potential to threaten students because it challenges their beliefs about themselves. Including this topic in the formal curriculum offends those who arrive at medical school feeling they already possess a wealth of expertise on this topic. It upsets those who regard themselves as fair and equitable in their treatment of others and feel this should be sufficient for medical encounters. It frustrates those who might recognize both unconscious and conscious bias in their peers but find themselves unable to speak up.

Our unsatisfying experience with the Culture TBL led us to explore the medical education literature in search of educational interventions that successfully engage learners in active learning experiences focused on the topic of culture as it pertains to healthcare. Perhaps unsurprisingly, we discovered others had also struggled to overcome the student perception of culture as ’soft science’ [8], and had heard some students ask ’why can’t we just treat a patient’s problem, not their race!!??’ [9]. Other authors had reported that their students denied the effects of race, class, gender, culture and sexual orientation on medical practice [10]. Other instructors had also had the painful experience of losing control of classroom discussions because of the emotional potency of issues around culture and difference [11].

Our literature review convinced us to change our educational approach. In August 2017 we retired the Culture TBL from the curriculum and replaced it with a large group discussion titled ‘Nutrition and Culture’, in which students practise taking a dietary history from ‘patients’ role-played by dietitians who portray different cultural backgrounds. We kept the reading that described the Kleinman and LEARN models, but replaced the other with a textbook chapter that omitted a reference list summarizing different ethnic cultures but encouraged readers to build their own insights into different cultures by interacting with patients and avoiding stereotypes [12]. This new Nutrition and Culture session successfully enabled a class discussion about cultural issues in patient care by challenging students to ask respectful questions about food choices in order to explore a patient’s cultural and personal beliefs. The class session was well received by students, nearly all of whom attended even though attendance was not required. As far as can be determined from their subsequent exam performance, it also helped students successfully master our pre-clerkship learning objectives for cultural awareness.

In retrospect, one of the most important lessons we learned from this experience is that different curricular topics call for different educational approaches. Having had previous success with converting per-clerkship lectures to TBLs, we simply failed to consider how the topic of culture might call for a different approach. In retrospect, we should have looked to the literature to guide us. When the emotional atmosphere surrounding a topic overwhelms the students, little learning can occur.

Moreover, it is now clear to us that an indirect approach to a topic might be the best educational strategy. Though we used student feedback to make iterative improvements in the TBL over several years, we ultimately decided to drop the patient vignettes that explicitly focused on race and culture. Instead, by asking students to take dietary histories in a role-play format, we successfully enabled conversations about culture in an emotionally ‘safe space’ where the overarching theme was food preferences and availability, dietary restrictions, and meal preparation.

In hindsight, we also recognize that persisting in a continuous quality improvement cycle ultimately did enable us to achieve our educational goals. Despite several years of struggle we finally succeeded both in emphasizing the cultural aspects of patient care early in the pre-clerkship curriculum and engaging students in active learning on this topic. Reverting to lecture format and excusing students from engaging in the discussion would have required less faculty effort. Nevertheless, we persisted in our efforts to create an active learning session and are glad we did not compromise on our goals.

Finally, although we could not have anticipated how history would unfold, we strongly suspect that events outside the classroom walls also prompted our students to become more open to considering how issues of race and culture might impact the experience of patients in in our healthcare system. After all, fierce and emotional conversations about race and culture have made national news since the 2016 election cycle. Subsequently, some patients have become more outspoken about their prejudices; in May 2017 the AAMC reported that physicians are struggling with being the target of bias [13]. In August 2017 our own university (University of Virginia) and progressive college town (Charlottesville) became sites of violent confrontations around topics of race and culture [14], shocking the university community as well as the citizenry of our city, state and country.

## Moral of the story

While it is tempting to attribute our recent classroom success entirely to innovative instructional design, the broader societal context undoubtedly influences how receptive students will be to some curricular lessons. Like students in other disciplines, medical students learn from their own experiences outside the classroom and not solely from carefully designed lesson plans. Medical educators must thoughtfully consider the content and delivery methods by which they introduce cultural topics into the curriculum because discussions about culture and race can provoke powerful emotional and unexpected responses from students that ultimately hinder learning. Nevertheless, if we are to ensure that all patients receive the highest quality medical care we must continue to prompt medical trainees to recognize and appreciate how cultural issues can impact health and disease, whatever the pedagogical challenges.

## References

[1] Association of American Medical Colleges (2005). Cultural competence education for medical students.

[2] Smedley B (2003). Unequal treatment: confronting racial and ethnic disparities in healthcare.

[3] Chen W, Worden M, Bradley E (2015). Flipping, engaging, and teaming, oh my! Lessons learned from a large scale curriculum reform at a US medical school.

[4] Michaelsen LK, Knight AB, Fink LD (2004). Team-based learning: a transformative use of small groups in college teaching.

[5] Berlin E, Fowkes WA (1983). A teaching framework for cross-cultural healthcare. West J Med.

[6] Kleinman A, Eisenberg L, Good B (1978). Clinical lessons from anthropologic and cross-cultural research. Ann Intern Med.

[7] Council of National Psychological Associations for the Advancement of Ethnic Minority Interests and the Association of Black Psychologists (2003). Psychological treatment of ethnic minority populations.

[8] Boutin-Foster C, Foster JC, Konopasek L (2008). Viewpoint: physician, know thyself: the professional culture of medicine as a framework for teaching cultural competence. Acad Med.

[9] Holmes D, Nettles R, Balter R (2012). ‘Why can’t we just simply treat people’s problems, not their race (or physical disability, or sexual orientation)!?’: a psychodynamic approach to the therapeutic relevance of multiple minority identities. Multiple minority identities: clinical, research, and educational implications.

[10] Beagan B (2003). Teaching social and cultural awareness to medical students: It’s all very nice to talk about it in theory, but it ultimately makes no difference. Acad Med.

[11] Willen SS (2013). Confronting a ‘big huge gaping wound’: emotion and anxiety in a cultural sensitivity course for psychiatry residents. Cult Med Psychiatry.

[12] Ball JW, Dains JE, Flynn JA, Solomon BS, Stewart RW (2015). Cultural competency in Seidel’s guide to physical examination.

[13] Warshaw R (2017). When the target of bias is the physician. AAMC news 2017.

[14] University of Virginia (2017). Charlottesville violence, August 11–12, 2017. Statements from the university community.

